# Fabrication of Composite Microneedle Array Electrode for Temperature and Bio-Signal Monitoring

**DOI:** 10.3390/s18041193

**Published:** 2018-04-13

**Authors:** Yiwei Sun, Lei Ren, Lelun Jiang, Yong Tang, Bin Liu

**Affiliations:** 1 School of Engineering, Guangdong Provincial Key Laboratory of Sensor Technology and Biomedical Instrument, Sun Yat-Sen University, Guangzhou 510006, China; sunshineyouve@163.com (Y.S.); renlei5@mail.sysu.edu.cn (L.R.); jianglel@mail.sysu.edu.cn (L.J.); 2 Guangdong Provincial Key Laboratory of Precision Equipment and Manufacturing Technology, South China University of Technology, Guangzhou 510640, China; ytang@scut.edu.cn

**Keywords:** microneedle array, composite electrode, body temperature, bio-signal

## Abstract

Body temperature and bio-signals are important health indicators that reflect the human health condition. However, monitoring these indexes is inconvenient and time-consuming, requires various instruments, and needs professional skill. In this study, a composite microneedle array electrode (CMAE) was designed and fabricated. It simultaneously detects body temperature and bio-signals. The CMAE consists of a 6 × 6 microneedles array with a height of 500 μm and a base diameter of 200 μm. Multiple insertion experiments indicate that the CMAE possesses excellent mechanical properties. The CMAE can pierce porcine skin 100 times without breaking or bending. A linear calibration relationship between temperature and voltage are experimentally obtained. Armpit temperature (35.8 °C) and forearm temperature (35.3 °C) are detected with the CMAE, and the measurements agree well with the data acquired with a clinical thermometer. Bio-signals including EII, ECG, and EMG are recorded and compared with those obtained by a commercial Ag/AgCl electrode. The CMAE continuously monitors bio-signals and is more convenient to apply because it does not require skin preparation and gel usage. The CMAE exhibits good potential for continuous and repetitive monitoring of body temperature and bio-signals.

## 1. Introduction

Recently, home health care management has attracted increasing attention given the rapid increase in the aging population. For elderly individuals living at home and especially paralysis patients and cerebral palsy patients, routine and repetitive measurement of a few basic physiological, including body temperature and bio-signals is important to reflect health conditions. Body temperature reflects the health condition of human beings and it is an indicator of a few diseases such as fever [1], insomnia [2], fatigue [3], metabolic functionality [4], and depression [5]. Bio-signals, including electrode-skin interface impedance (EII), electromyography (EMG) and electro-cardiography (ECG) are also crucial in clinical practice applications such as the detection of heart attacks and rhythm disorders by using a ECG signal, identification of epilepsy by using a EEG signal, and monitoring of neurogenic myopathy by using a EII signals [6]. The combination of body temperature with bio-signals provides clinically relevant information related to cardiovascular health, cognitive state, malignancy, and many other important aspects of human physiology.

Clinical mercurial thermometer (CMT), ear thermometers, and infrared digital cameras are usually employed for body temperature measurements. Oral, rectal, and organ (tissue) temperature measurements with a clinical thermometer or an ear thermometer provide more accurate readings of body temperature although these techniques are limited to single-point and non-continuous measurements. Furthermore, the use of a clinical thermometer is time consuming. An infrared digital camera exhibits advantages including quick, responsible, and non-contact detection although it is limited by measurement accuracy in clinical applications. Recently, a few wearable devices integrated with a thermometer were developed for real time monitoring of skin temperature [7,8,9,10]. Skin temperature is easily affected by surrounding temperature and humidity. Therefore, it is necessary to consider accurate detection and long-term monitoring of body temperature for home health care management.

Ag/AgCl electrodes and dry electrodes are the most widely used electrodes to detect bio-signals. Specifically, the Ag/AgCl electrode suffers from two major defects, namely skin preparation and the use of gel. A microneedle array electrode is a promising dry electrode that has attracted increasing attention in EII [11], ECG [12], EMG [13], and EEG [14] monitoring due to excellent properties including painless puncture without skin preparation, minimal skin trauma, reduced infection, ease of operation, and high selectivity [15,16]. The previous microneedle array electrode mainly focused on the bio-signals recording. No report has been found that a microneedle array electrode can simultaneously monitor both bio-signals and body temperature.

In this study, a novel composite microneedle array electrode (CMAE) was fabricated for body temperature and bio-signals monitoring. The microneedle penetrated the skin through the stratum corneum layer to efficiently detect body temperature and bio-signals. The fabrication method of the CMAE is simple and incurs low cost. The fabrication comprises three steps, namely fabrication of a titanium microneedle array, spinning coating of a SU-8 layer, and sputtering coating of a gold layer. The measuring mechanism of the CMAE is discussed. The fabricated microneedle was characterized, and its mechanical performance was investigated under multiple insertions. The temperature measurement performance of the CMAE is discussed. The performance of monitoring EII, EMG, and ECG bio-signals was evaluated and compared with those of commercial Ag/AgCl electrodes.

## 2. Experimental

This study was approved by the ethics committee of the Work Injury Rehabilitation Center of Guangdong Province (approval number: AF/SC-07/2016.29). All volunteers provided informed written consent.

### 2.1. Fabrication of the CMAE

The fabrication process of CMAE consisted of three steps, namely the fabrication of a microneedle array, spinning coating of an insulating layer, and sputter coating of a gold layer:

(1) Fabrication of microneedle array. A 6 × 6 microneedle array was cut on a titanium alloy sheet (TC4, thickness of 100 μm, Baoji Titanium Industry Co., Ltd., Baoji, Shaanxi, China) by a focused beam laser with a pulse fiber laser machine (IPG, No.: YLP-1-100-20-20-CN, Burbach, Germany) as described previously [17]. The laser power was 15 W, laser frequency was 20 kHz, scanning speed was 500 mm/s, and scanning number was 50. The titanium sheet was completely cut through to form microneedles in a plane. These microneedles were manually bent 90° out of the plane. A microneedle array (MNA) was formed.
(2) Spinning coating of the insulating layer. The MNA was assembled in a vacuum spinner (VTC-100, MTI Corporation, Richmond, CA, USA). 2 mL of SU-8 solution (Formlabs Inc., Somerville, MA, USA) was dropped on the upper surface of the MNA at 500 r/min. The MNA coated with the SU-8 layer was cured in a UV ultraviolet curing machine (Intelli-Ray 400, Uvitron, West Springfield, MA, USA) at 400 W for 10 min.
(3) Sputter coating of Au layer. 100 nm thickness Au film was coated on the surface of the micro-needle array by a magnetron sputtering machine (VTC-16-SM, Shenyang Kejing Auto-instrument Co., Ltd., Jilin, China). A titanium line and a gold line were welded to the titanium layer and the gold layer, respectively. The CMAE was fabricated.

The CMAE was observed by scanning electron microscopy (SEM, JSM-6380LA, JEOL, Tokyo, Japan).

### 2.2. Mechanical Performance Test of the CMAE

Multiple insertion tests were used to assess the mechanical and stability properties of the CMAE and to determine their ability to repeat insertions in body temperature and bio-signal monitoring. Porcine cadaver skin was used for the insertion tests. The hair of the porcine cadaver skin was shaved off by a razor, and the subcutaneous fat was removed by a scalpel [18]. The skin was cut into 5 cm × 10 cm squares with a thickness of 1.5 mm. The skin was pre-tensed and fixed on a wooden plate by using pins.

The insertion test was performed on a universal material testing machine (LR10K Plus, Lloyd Instruments, Bognor Regis, UK). The experimental procedures were as follows: (1) The CMAE dipped with red ink was bonded on the upper compression plate of the testing machine, and the porcine cadaver skin was fixed on down compression plate. (2) The CMAE moved down and pierced into the skin at a speed of 0.01 mm/s. (3) The test was stopped when the loading force reached 10 N. The CMAE was drawn back. (4) In order to verify the reliability of the CMAE, the above procedures were repeated 100 times.

The punctured porcine skins were observed with a digital camera (5D III, Canon, Tokyo, Japan). The intact microneedle percentage (the number of red spots divided by the total number of microneedles per array) was calculated.

### 2.3. Temperature Measurement

**Figure 1:**
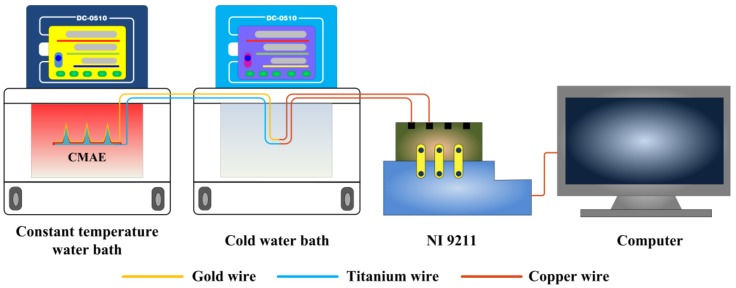
Schematic of the temperature calibration experimental setup.

It was necessary to first calibrate the CMAE prior to the actual measurement. A calibration system was self-developed as shown in Figure 1 [19,20]. It was mainly composed of two water baths (DC-0510, Ningbo Scientz Biotechnology Co., Ltd., Ningbo, China), a data acquisition card (NI9211, National Instruments Corporation, Austin, TX, USA), and a computer. The water baths were used as the heat source and the cold source, respectively. The temperature range of the water baths was from −5 °C to 100 °C with a control accuracy of ±0.05 °C. A data acquisition card and a LabVIEW acquisition program in a computer were employed to collect the temperature and voltage signals.

The electrical characterization of the CMAE was calibrated by measuring the output voltage and the setting temperature. The CMAE and the cold junction were dipped into a constant water bath and a cold water bath. The voltage was measured at the cold junction point. The temperature of the constant temperature bath gradually increased from 25 °C to 50 °C with an increment of 5 °C. The temperature of cold bath was maintained at 0 °C. The voltage generated by CMAE at a steady state was collected. The calibration procedures were repeated thrice.

Body temperature measurements were conducted on a 29-year-old healthy male subject. The CMAE were placed on the forearm and armpit to detect the body temperature. The testing result was automatically collected by the data acquisition system. A standard clinical mercury thermometer (Yuwell, Jiangsu Yuyue Medical Equipment & Supply Co., Ltd., Danyang, China) was also used to detect the body temperature and compared with the result obtained with the CMAE. The average skin moisture content is approximately 30% which was measured by a commercial moisture meter (Delfin Moisture Meter SC, Kuopio, Finland). The body temperature measurements were repeated thrice.

The temperature measurements were performed in a clean room to investigate the effects of surrounding temperature and humidity on the temperature measurement of CMAE. Firstly, the temperature was set at 22 °C, and then the air conditioner was opened. The room temperature was increased from 22 °C to 29 °C and maintained stable for approximately 20 min. A dehumidifier was used to decrease the relative humidity from 76% to 52% and kept stable at 52%. The ambient temperature and relative humidity were measured with a commercial Sensirion sensor.

### 2.4. Bio-Signals Recording

Additionally, EII, EMG, and ECG recording tests were performed with both the CMAE and commercial Ag/AgCl electrodes (JK-1(A-H) type Shanghai Junkang Medical Supplies Ltd., Co., Shanghai, China) to better understand the bio-signal recording performance of CMAE. Tests were performed on a 25-year-old female volunteer after obtaining the subject’s informed consent. The average skin moisture content is approximately 30%. The measurement was first performed with the CMAE and then repeated with Ag/AgCl electrodes.

#### 2.4.1. EII Test

Two-electrode measuring methods [21] were employed to measure the EII with the CMAE and the Ag/AgCl electrode. The inner forearm was selected as the measurement place given that it exhibits less hair and a thinner stratum corneum and is convenient for the placement of electrodes [11]. Two electrodes were firmly packed on the left forearm of the volunteer with an interval of 5 cm, and the distance between the right electrode and wrist was also 5 cm [22]. Subsequently, the electrodes were connected to a precision impedance analyzer (E4980A LCR Meter, Agilent, Palo Alto, CA, USA). All EII signals were continuously recorded with an input voltage frequency ranging from 20 Hz to 10 kHz. The EII test result of the CMAE was compared with that of the Ag/AgCl electrode. All measurements were performed in ambient conditions with an approximate temperature of 25 °C and humidity of 60%.

#### 2.4.2. EMG Test

The biceps brachii muscle activity was analyzed with the CMAE and Ag/AgCl electrode with respect to flexion of the elbow of the right hand. Two measuring electrodes were placed on the biceps brachii muscle with an interval of 2 cm [23], and the grounding electrode (Ag/AgCl electrode) was attached on the elbow. Signals were acquired with a EMG100C module of a multipurpose polygraph (MP150, BIOPAC, Goleta, CA, USA). The test lasted for 5 min. During the test, the amplifier gain was set at 2000, and the sample rate was set at 1000 Hz. A 20 Hz–450 Hz Butterworth band-pass filter was used.

#### 2.4.3. ECG Test

The ECG was recorded by a standard II-lead method. The performance of CMAE was compared with that of standard Ag/AgCl electrodes. Additionally, measuring electrodes (CMAE) were stuck on both wrists of the volunteer, and the grounding electrode (Ag/AgCl electrode) was placed on the right ankle. Three electrodes were connected to the ECG100C module of a multipurpose polygraph (MP150, BIOPAC). The volunteer lay on a bed during the ECG recording. The test lasted at least half an hour. During the test, the amplifier gain was set at 5000, the sample rate was set at 1000 Hz, the high-pass filter was set at 0.5 Hz, and the low-pass filter was set at 35 Hz LPN.

## 3. Results and Discussion

### 3.1. Measure Mechanism of the CMAE

**Figure 2:**
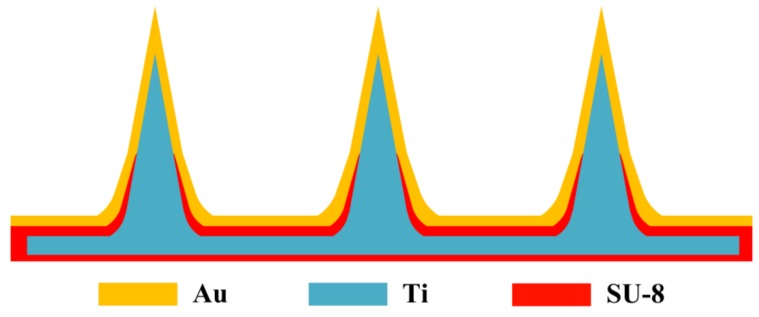
Structural diagram of the CMAE.

A composite microneedle array electrode was designed, and its structural diagram is shown in Figure 2. The CMAE was composed of three layers, namely the titanium layer, SU-8 layer, and gold layer. The microneedles were fabricated from the titanium layer. The SU-8 film was spin coated on the titanium layer. The gold layer was sputter coated on the SU-8 layer. The titanium and the gold layer were in contact with each other on the tip of the microneedle array. A gold wire and a titanium wire were connected to the gold layer and titanium layer, respectively. The CMAE was an electrical device that consisted of two dissimilar electrical conductors (titanium and gold) forming electrical junctions at different temperatures. The SU-8 layer was used to insulate the titanium and gold. It produces a temperature-dependent voltage due to the thermoelectric effect, and the voltage is interpreted to measure the temperature.

A CMAE penetrates through the SC layer and gold layer and is directly in contact with the skin tissue. It converts the weak ion current of the human body to an electrical signal [15,24].

**Figure 3:**
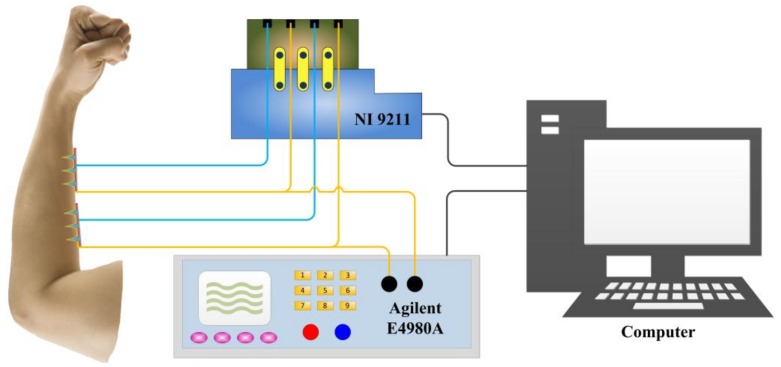
Comprehensive detection of EII and temperature with the CMAE.

Figure 3 shows the schematic illustration of a combination of EII recording and temperature monitoring by CMAE on the forearm. The temperature was measured with gold layer and titanium layer of the CMAE and acquired by NI9211. The EII signal was measured by using gold layers of two CMAEs and collected by a precision impedance analyzer. All the data were analyzed using a computer.

### 3.2. Characterization

**Figure 4:**
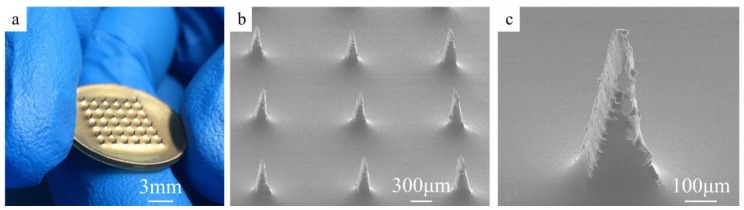
(**a**) Photograph of the CMAE; (**b**) SEM image of the CMAE; (**c**) SEM image of a single microneedle.

The image of CMAE is shown in Figure 4a. A 6 × 6 microneedle array was neatly arranged. Furthermore, Au films were uniformly coated on the surface of the CMAE, and the thickness of the gold was approximately 100 nm. The coated Au film guaranteed the conductivity and biocompatibility of the CMAE. The titanium and gold were selected for their excellent biocompatibility properties. Additionally, the SU-8 was used to fabricate microneedle arrays [25,26] in a few previous medical studies. Therefore, the materials were safe for clinical use. The SEM image of the CMAE is shown in Figure 4b. The average height of a microneedle is 500 µm, the equivalent diameter of the microneedle is 200 µm, and the interval between adjacent microneedles is 1.2 mm. The thicknesses of the corneum layer and the stratum germinativum layer are about 15–20 µm and 150–180 µm, respectively [23]. In most case, only about 1/3 of the overall height of the microneedles could penetrate in the skin [21,23]. Therefore, 500-µm microneedle height of CMAE was appropriate for bio-signal recording. Additionally, the dimensions of the CMAE were adjusted with the design and fabrication process based on different applications. Further investigations of the SEM image revealed small humps at the bottom of the microneedles due to the spin coating of the SU-8, and this may be beneficial for the mechanical properties of the CMAE. Figure 4c illustrates the SEM image of a single needle. The micro-needle tip is sharp, and this is helpful in reducing the piercing force and the feeling of pain experienced by individuals during the piercing process [27,28]. The surface of the CMAE is rough due to the laser fabrication process, and this increased the contact area and stability between CMAE and skin [11].

### 3.3. Mechanical Properties

**Figure 5:**
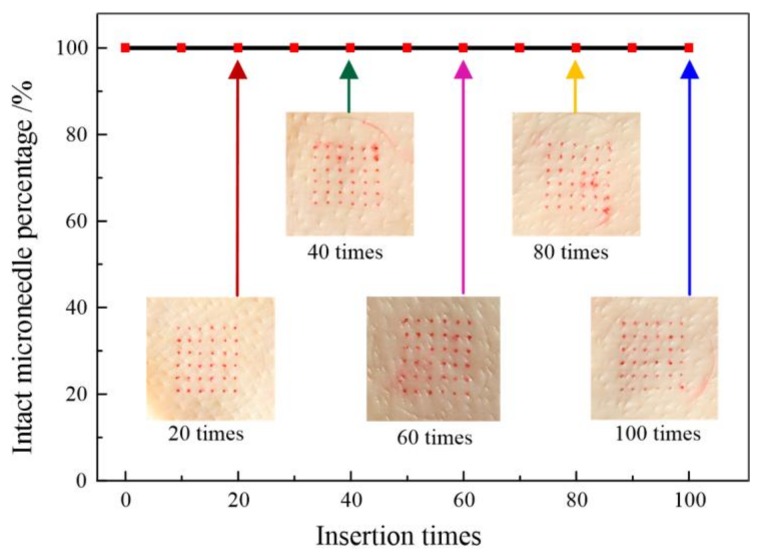
Relationship between intact microneedle percentage and insertion times.

With respect to the proposed microneedles in bio-signal monitoring, the microneedle array should be sufficiently strong to penetrate the skin without breaking [27]. It is necessary for the CMAE to penetrate the skin without breakage. Therefore, it is necessary to investigate multiple insertions and especially the repeated usage ability of microneedles. The mechanical stability of the CMAE was evaluated by the relationship between the percentage of successful insertions and the number of MNs on a patch. Figure 5 shows the relationship between intact microneedle percentage and insertion times. The red dots remaining on the surface of the skin were well-arranged in a 6 × 6 array and coincided with the microneedle array arrange. It indicates that the CMAE pierced the skin, and this implies the successful insertion of the microneedle. The red dots on the skin were counted to calculate the intact microneedle percentage per array as shown in the top line of Figure 5. Specifically, 100% successful insertion indicates the initial excellent mechanical properties of the CMAE. The intact microneedle percentage remained at 100% with further increases in the insertion numbers to 100 times. However, the percentage decreased to 90% after 10 pierces with 600 µm high polymer microneedles as reported by Li et al. [18]. Additionally, the skin after 100 insertions was almost the same as that after the first insertion. This demonstrated that the CMAE was sufficiently strong and reliable to penetrate the skin 100 times without exhibiting damage or bending. It indicates that it is possible to repeatedly use the CMAE in body temperature and bio-signal monitoring and that the issue of broken microneedles was resolved.

### 3.4. Temperature Measurement Performance of the CMAE

**Figure 6:**
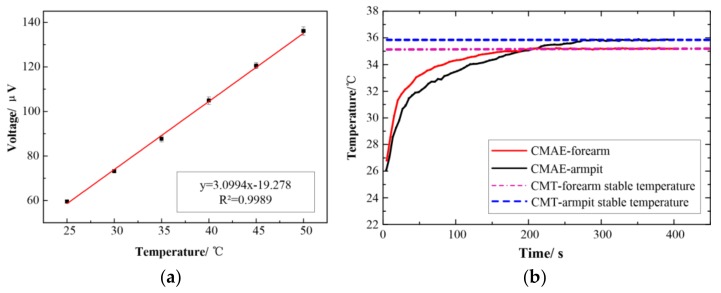
Temperature measurement performance of the CMAE (**a**) Temperature calibration curve; (**b**) Temperature measurement curve.

Figure 6a shows the results of temperature calibration curve, good linearity was clearly observed in the range of body temperature. The fitting line expression is:

y = 3.0994x − 19.278(1)

where y is the voltage, and x is the temperature.

The coefficient of determination of the line was 0.9989. It indicated that the linear relation between the temperature and voltage was favorable. The error bar of the repeated measurement was potentially due to the slight variation in the temperature at cold junction. The excellent linear relationship ensures that the CMAE accurately detected the body temperature. The experimental Seebeck coefficients of the CMAE corresponded to 3.09 µV/°C, and this was in the same order of magnitude as 7.71 µV/°C of a Pt–10%Rh/Pt thin film thermocouple [19] and 8.87 µV/°C of a thin film thermocouple [29].

Figure 6b shows the results of body temperature measurement obtained with the CMAE on the forearm and under the armpit of a 29-year-old male subject. The steady temperature of armpit was 35.8 °C by CMAE, and this was the same as that obtained by using the CMT. This indicated that the CMAE obtained an accurate core temperature. The steady temperature of the forearm was 35.3 °C as measured by the CMAE and 35.2 °C as measured by the CMT. Therefore, the forearm temperature by CMAE is slightly higher, and this indicated that the CMAE pierced into skin to measure the core temperature. The startup time of the CMAE is approximately 5 min, and this is in the same level with clinical thermometer which also needs 5 min to reaches the stable stage. Most importantly, it indicates good potential in real-time and long-term measurement of body temperature.

**Figure 7:**
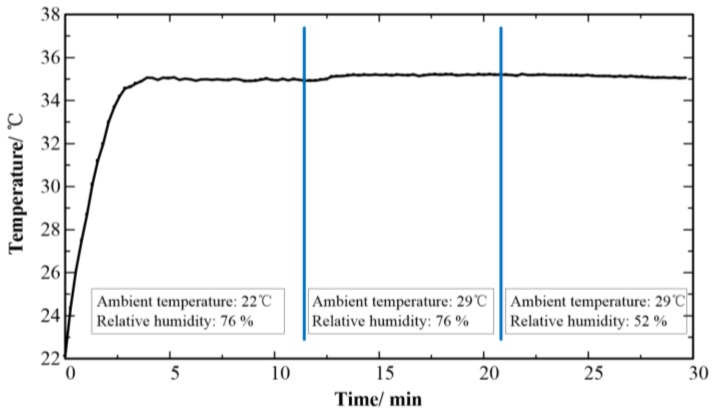
Effects of surrounding temperature and humidity on temperature detection of CMAE.

Figure 7 shows the results of body temperature measurement obtained with the CMAE on the forearm under different surrounding environment. The body temperature is approximately 35 °C at ambient temperature of 22 °C and relative humidity of 76%. The body temperature increases to 35.1 °C as the room temperature is increased at 29 °C. The body temperature varies little as the skin humidity decreases from 76% to 52%. Therefore, ambient environment effects little on the temperature measurement results using CMAE.

### 3.5. Performance of the CMAE for Recording Bio-Signals

#### 3.5.1. EII Measurement

**Figure 8:**
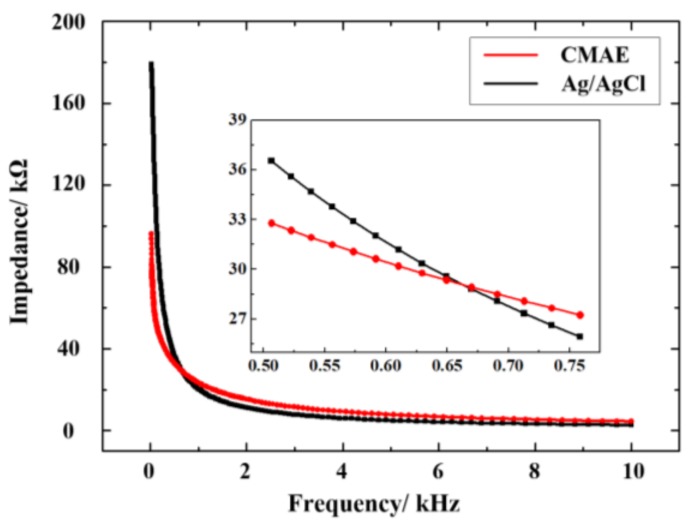
Impedance curve of the CMAE and Ag/AgCl electrode.

The EII measured by CMAE and Ag/AgCl electrode at a driving current frequency from 20 Hz to 10 kHz are shown in Figure 8. The EII consists of resistance and capacitive resistance, and the capacitive resistance decreases the frequency. The impedance measured by CMAE was lower than that by Ag/AgCl electrode at a low frequency. This was potentially because the CMAE pierced through the stratum corneum on the skin and reduced the contact impedance [30]. The impedance measured by CMAE gradually exceeded that measured by the Ag/AgCl electrode with increases in the frequency. The Ag/AgCl electrode is usually used with a conductive gel or paste and requires skin preparation, and this is time-consuming and may lead to a few infections on the skin [24]. The surface of the Ag/AgCl electrode is about 100 mm^2^ and the surface of the CMAE is about 16 mm^2^. The surface of the Ag/AgCl electrode is about 6 times larger than the CMAE. It is significantly more convenient to use the CMAE as it does not require skin preparation and the use of a gel [31]. Therefore, the CMAE can be used for EII monitoring.

#### 3.5.2. EMG Measurement

**Figure 9:**
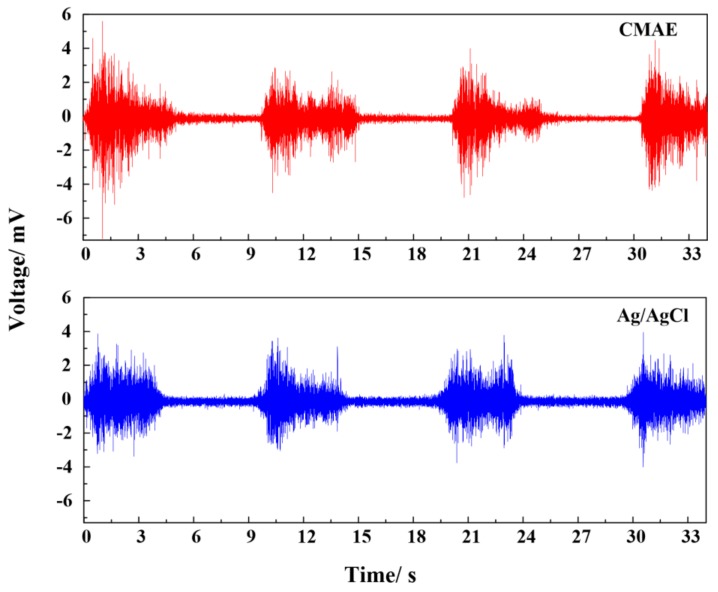
EMG signals recorded by CMAE and Ag/AgCl electrode.

Figure 9 clearly reflects the EMG signals recorded by CMAE and Ag/AgCl electrodes. The signals measured by CMAE and Ag/AgCl electrodes fluctuate periodically relative to the motion of the biceps brachii muscle. When the muscle contracts from the relaxation state, the muscle cells are electrically or neurologically activated, and the electrical potential increases rapidly to a high amplitude. Otherwise, the voltages decrease to a low level. It was observed that signals acquired with the two different types of electrodes were very similar in shape and amplitude. The amplitude of the CMAE slightly exceeded that of the Ag/AgCl electrode. This measurement again demonstrated the ability of CMAE to sense and record surface biopotential EMG signals with good fidelity. According to the data acquired with the CMAE and Ag/AgCl electrodes, the RMS data acquired with the CMAE was 0.6417, which was higher than 0.5681 measured by Ag/AgCl electrodes. It indicated that the level of muscle activation was higher as the CMAE was applied. The contact surface of the CMAE is lower in size than that of standard Ag/AgCl electrodes [30], and thus CMAE is more convenient to improve the selectivity of EMG signal and to reduce the crosstalk. This result may be helpful in a few critical situations when several muscles are present in small spaces or when subjects, such as children, exhibit low anthropometric dimensions. Therefore, CMAE is a good choice for EMG recording without skin preparation when compared with Ag/AgCl electrodes.

#### 3.5.3. ECG Measurement

**Figure 10:**
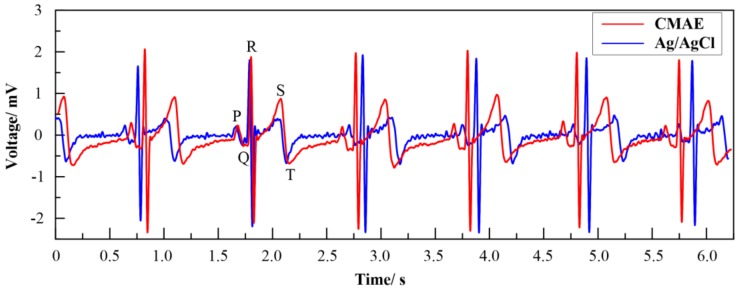
ECG signals recorded by the CMAE and Ag/AgCl electrode.

Specifically, the ECG signals recorded by CMAE and Ag/AgCl electrode are shown in Figure 10. The recording performance of the CMAE was comparable to those of conventional Ag/AgCl electrodes. Typical cardiac signatures including P-wave, QRS-complex, and T-wave acquired by the CMAE were clearly visible. Additionally, the amplitude slightly exceeded that of the Ag/AgCl electrode. The result indicated good agreement with those obtained in previous studies [12,32]. This indicates that the CMAE exhibits the ability to detect ECG signals. Thus, the CMAE exhibits a potential to routinely and repetitively measure the ECG.

## 4. Conclusions

In this study, a novel CMAE was designed and fabricated to simultaneously record body temperature and bio-signals. It consisted of three layers, namely a titanium microneedle array layer, a SU-8 insulating layer, and a gold layer. A 6 × 6 microneedle array was neatly arranged. The height of the microneedle was 500 µm, and the equivalent diameter of the microneedle base was 200 µm. The CMAE exhibited excellent mechanical properties, including the ability to be inserted in the skin 100 times without breaking. The CMAE exhibited good temperature monitoring performance since it accurately monitored the body temperature when compared with a clinical thermometer. The EII recorded by the CMAE was lower in the range of 20 Hz–0.675 kHz when compared with that of a Ag/AgCl electrode. The EMG signals recorded by the CMAE and Ag/AgCl electrode exhibited similar shape and amplitude. The typical P-wave, QRS-complex, and T-wave of an ECG signal recorded by the CMAE were distinguishable. The CMAE was easy to use since it does not require skin preparation, and thus it is more comfortable and convenient for patients. Therefore, the CMAE exhibits good potential for use in monitoring body temperature and bio-signals.
